# Assessment of Feasibility to Use Computer Aided Texture Analysis Based Tool for Parametric Images of Suspicious Lesions in DCE-MR Mammography

**DOI:** 10.1155/2013/872676

**Published:** 2013-04-09

**Authors:** Mehmet Cemil Kale, John David Fleig, Nazım İmal

**Affiliations:** ^1^Bilecik Şeyh Edebali University, Turkey; ^2^Franklin University, OH, USA

## Abstract

Our aim was to analyze the feasibility of computer aided malignant tumor detection using the traditional texture analysis applied on two-compartment-based parameter pseudoimages of dynamic contrast-enhanced magnetic resonance (DCE-MR) breast image data. A major contribution of this research will be the through-plane assessment capability. Texture analysis was performed on two-compartment-based pseudo images of DCE-MRI datasets of breast data of eight subjects. The resulting texture parameter pseudo images were inputted to a feedforward neural network classification system which uses the manual segmentations of a primary radiologist as a gold standard, and each voxel was assigned as malignant or nonmalignant. The classification results were compared with the lesions manually segmented by a second radiologist. Results show that the mean true positive fraction (TPF) and false positive fraction (FPF) performance of the classifier vs. primary radiologist is statistically as good as the mean TPF and FPF performance of the second radiologist vs. primary radiologist with a confidence interval of 95% using a one-sample *t*-test with *α* = 0.05. In the experiment implemented on all of the eight subjects, all malignant tumors marked by the primary radiologist were classified to be malignant by the computer classifier. Our results have shown that neural network classification using the textural parameters for automated screening of two-compartment-based parameter pseudo images of DCE-MRI as input data can be a supportive tool for the radiologists in the preassessment stage to show the possible cancerous regions and in the postassessment stage to review the segmentations especially in analyzing complex DCE-MRI cases.

## 1. Introduction

Dynamic contrast-enhanced magnetic resonance imaging (DCE-MRI) has become an important imaging approach for the evaluation of microcirculation in tumors [1]. It is becoming a capable noninvasive method to monitor tumor response to therapies; however, current practice requires radiologists to manually identify and segment tumors from the imaging data. Frequently used methods of identifying tumors on DCE-MR images are based on pseudoimages of parameters obtained by locally fitting time-intensity curves to a two compartment exchange model for contrast agent concentration [2] or use the three time point method [3]. These approaches are very tedious, time consuming, and prone to intra- and interobserver variation. 

Texture analysis has been applied in medical imaging for several applications. Tourassi described the role of image texture analysis in the medical imaging field [4]. Lerski et al. used texture analysis on MRI for tissue characterization and segmentation of brain tissues [5]. Kovalev et al. implemented a method for object recognition and matching with multidimensional cooccurrence matrix calculations [6–8]. Gibbs and Turnbull used a cooccurrence-based texture tool on postcontrast MR breast images, showing that there is a difference in terms of the spatial variations in voxel intensities between benign and malignant lesions [9].

Neural networks are widely used for image classification [10]. Some examples of neural network classification on MRI prior to our method are briefly presented here. Vergnaghi et al. used an artificial neural network to automatically classify the enhancement curves as benign or malignant [11]. Lucht et al. described a voxel-by-voxel neural network classifier which compared the performance of quantitative methods for the characterization of signal-time curves acquired by DCE-MR mammography [12–14]. Tzacheva et al. implemented a region-based neural network classifier which segments malignant tissues using a static postcontrast T1-weighted image as input [15]. Szabó et al. implemented an artificial-neural-network- (ANN-) based segmentation method for DCE-MRI of the breast and compared it with quantitative and empirical parameter mapping techniques to test the discriminative ability of kinetic, morphologic, and combined MR features [16, 17]. Twellmann et al. introduced a model-free neural network classifier where he suggested that an improvement could likely be achieved using texture features [18].

We present a computer aided diagnostic (CAD) tool based on statistical texture voxel-by-voxel analysis of pseudoimages generated by the local curve fitting of parameters from a two compartment exchange model. The texture processing is based on statistical methods originally presented by Haralick et al. [19]. The CAD tool we describe in this paper is a texture-based classifier which uses two-compartment-based DCE-MRI breast datasets. Compared to the studies of Gibbs and Turnbull, the work reported here focuses on the parameter pseudoimages derived from curve fitting of the two-compartment model from the original MRI dataset. Classification of the outcome images by the texture-based analysis tool is obtained using an artificial neural network trained with data marked by a radiologist.

## 2. Materials and Methods

### 2.1. Data Acquisition

The two-compartment model for contrast agent exchange is the basis for the work presented here [20]. In this model, the primary compartment represents intravascular space, with extravascular (extracellular) space as the secondary compartment. Typically the two-compartment model is characterized by the following parameters. The first-order rate constant for transfer from the primary (vascular) compartment to the secondary (extravascular, extracellular) is *k*_pe_. The rate constant in the reverse direction is *k*_ep_. Injection flow rate for the contrast agent is *k*_in_, and the first-order rate constant for elimination of the contrast agent from the primary compartment is *k*_el_. The peak intensity reached for a given voxel during the time course of the DCE-MRI acquisition is *A*. 

To identify voxels representing malignant tissues, radiologists rely on the property that microvessels in malignant tissues are more frequent and porous than normal. This difference is represented in the local parametric values of *A*, *k*_ep_, and *k*_el_, which are used by the reader to mark voxels and regions as malignant from color-coded pseudoimages that combine ranges of values of these local parameters [2]. Since these parameters are useful for human classification, we concentrated on the spatial statistics of these three parameters for the texture analysis in the development of the CAD tool. 

**Figure 1:**
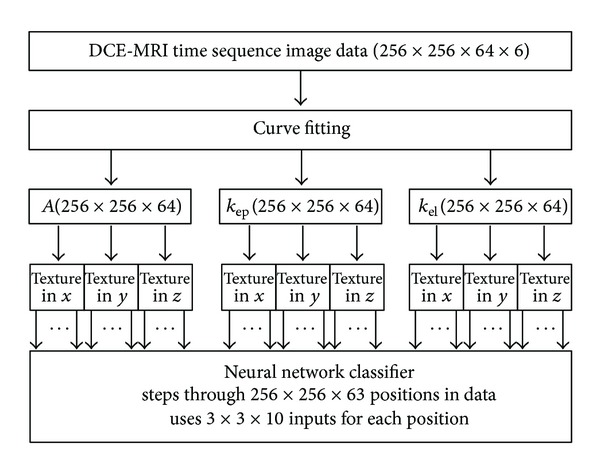
Flow diagram of the computer aided tool.

For this study, each pseudoimage dataset consisted of six-time samples of a 64 slice stack of 256 × 256 images. Images were acquired as a set of coronal slices using 1.5 T MRI scan (Magnetom Vision; Siemens, Erlangen, Germany) with extracellular Gd-chelate contrast agent in a dosage of 0.2 mL/kg bodyweight for a constant bolus injection duration of 7 seconds. Using dynamic T1-weighted gradient echo sequence (TR = 8.1 ms, TE = 4.0 ms, flip angle = 20 degrees), 64 coronal slices were produced where each slice consisted of 256 × 256 pixels, an effective slice thickness of 2.5 mm, and a field of view of 320 × 320 mm^2^. For six different time points, a volume dataset was acquired. The first volume dataset was the baseline intensity, and the following five volume scans were taken 120 seconds apart to monitor the exchange rate and elimination of the contrast agent. DCE-MR images obtained with a temporal resolution of less than 15 seconds have been shown to seriously underestimate pharmacokinetic parameters. Using time sequences of MR images, a time-intensity curve was constructed for each voxel in a DCE-MRI dataset and matched to a curve representing the two-compartment exchange model for contrast agent using a curve-fitting algorithm described in the literature [2]. For each DCE-MRI case, we calculated three separate 256 × 256 × 64 parameter pseudoimage sets, one for *A*, one for *k*_ep_, and one for *k*_el_. The flow diagram of the method beginning from the construction of these parameter pseudoimage sets is described in Figure 1. 

### 2.2. Radiologist Assessments of Subjects

Two radiologists analysed the images. The primary radiologist was used as the gold standard, and the second radiologist was used for the observation of the differences between the radiologist assessments and comparison of these with the differences between the computer classifier versus primary radiologist. Images were marked using a pharmacokinetic two-compartment-based analysis software tool implemented in IDL (Interactive Data Language, Boulder, Colorado, USA). The estimated pharmacokinetic parameters *A* and *k*_ep_ were color-coded and superimposed on the precontrast MR image. The radiologists then manually segmented the lesions using a computer mouse. Although some of the lesions had both malignant and benign components [21, 22], lesions were labeled either as malignant or benign.

### 2.3. Statistical Cooccurrence Analysis

The general approach of cooccurrence based texture analysis is to examine the signal values of pixels compared with one another in particular configurations. This approach starts with the assumption that we are observing a particular subset of an image, such as an *M* × *N* × *L* scanning volume of interest (SVOI). A joint histogram may be described by a matrix of relative frequencies of occurrence *P*_*θ*,*d*_(*a*, *b*), describing how frequently two pixels with gray levels *a* and *b* appear in the window separated by a distance *d* (chessboard distance measurement) in a given direction angle *θ* [23, 24]. This matrix is called the cooccurrence matrix. In our studies, we used distance *d* = 1 and angle *θ* to be three principal directions in the 3D Cartesian coordinate system for the original image data. In our approach, we use the local values of *A*, *k*_ep_, and *k*_el_ instead of the original gray levels of the MR image.

A separate 2-variable (2-V) cooccurrence matrix of each parameter is developed by using statistical cooccurrence texture analysis. The analysis is based on three-dimensional (3D) SVOIs. The dimension of the SVOI used in this study is 5 × 5 × 2, with the shorter length in the through-plane direction. The 5 × 5 × 2 window is raster scanned through each set of pseudoimages (*A*, *k*_ep_,  and *k*_el_) in the 3D Cartesian coordinate system and at each step in the raster scan, a cooccurrence matrix is formed by observing relative parameters of neighboring voxels in a given direction within the current location of the SVOI.

After the formation of the 2-V normalized cooccurrence matrix, for each of the DCE model parameters (*A*, *k*_ep_, *k*_el_) we computed ten types of statistical properties described by Haralick et al. [19]: angular second moment, correlation, contrast, inverse difference moment, variance, sum average, sum variance, sum entropy, entropy, and difference variance. Difference entropy was not used in our experiments because of its similarity to sum entropy. Maximal correlation coefficient parameters were also not used in our experiments because they involve a singular value decomposition which can be computationally unstable if the cooccurrence matrix is ill conditioned. Information correlation measurements were excluded from our set of used parameters. Although they have some desirable properties which are not contained in the rectangular correlation parameter, these parameters did not improve the results. The values of the calculated statistical properties were assigned to the spatial location of the current centroid of the SVOI. In this fashion, we constructed ten new parametric pseudoimage datasets associated for each pharmacokinetic parameter of the DCE-MRI case (total of 30 new pseudoimage datasets). Including separate texture properties for each of the three cardinal directions through 3-D space, we calculated a collection of 90 3-D local texture property pseudoimages for each subject. Because of the size of the data used and to make the execution time reasonable, we used a distributed and parallel environment middleware, image processing for the grid (IP4G) which has been developed for image analysis applications in a grid environment [25]. 

### 2.4. Neural Network Classifier

For computer classification, we used a voxelwise feedforward neural network classifier with purelin back-propagation using the 90 different reconstructed texture property pseudoimages as the set of inputs to an artificial neural network. The neural network classifier was based on the weighted combinations of textural parameter inputs which minimized the mean square error (MSE). The training set was derived from the voxels of a subset of image slices picked for the training set. This weighted sum was applied on the testing set composed of the remaining voxels of the pseudoimage slices picked for the training set and all voxels of the other image slices. The neural network had one hidden layer having 15 neurons and an output layer having 1 neuron. Using one hidden layer gave the best results.

Eight subjects verified by pathology were used in the study. Six subjects (A, C, D, F, G, and H) had IDC breast tumors, and two subjects had benign lesions (B, E). In the experiment six image slices from three subjects (subjects B, C, and D) were used to construct the training sets. Picking a training set in this manner included a mixture of tumor types.

In generating the training set, parenchymal combination of tissues and tissues marked as benign or malignant by the primary radiologist were used. First, half of the voxels of the malignant or benign tissues marked by the primary radiologist were picked, and the coordinates of these voxels were assigned to the training set. Then, same number of voxels was picked randomly from the body tissue. The coordinates of these randomly picked voxels were also assigned to the training set. A collection of verification voxels were chosen in the same manner as the training set to prevent overtraining of the neural network. Half of the voxels picked for the training set were assigned to the verification set, and those chosen voxels were excluded from the training set. In our experiments, there were 1129 voxels in the training set and 1128 voxels in the verification set. The selection of similar populations in the two classes is a common strategy in training artificial neural network classifiers which allows the computer classifier neural network to understand different types of tissues.

For the study, the cancerous regions marked by the primary radiologist were used as the ground truth to assess voxelwise true positives, false positives, true negatives, and false negatives in comparing the performance of a second radiologist with the computer classifier relative to the ground truth. In this comparison, voxels used in the training set were eliminated from the reported comparison results to avoid a bias. For each voxel studied, the neural network produced an output ranging from 0 to 1, which was thresholded to classify the voxel content as malignant or nonmalignant. Thresholds were varied to observe the voxelwise rate of false positives, true positives, false negatives, and true negatives to generate a scatter plot for the classifier. The threshold levels used in our experiments were 39%, 49%, 59%, 69%, 78%, 88%, and 98%.

### 2.5. Comparing the Performance of Computer Classifier with the Second Radiologist

A statistical comparison of the results of the classifier and the second radiologist was done by paired one-sample *t*-test described by Rosner [26]. Two different tests were applied, where the first test compared the true positive fraction (TPF) performances of the computer classifier and the second radiologist, and the second test compared the false positive fraction (FPF) performances of the computer classifier and the second radiologist. In order to perform a paired test, we formed a difference TPF vector and a difference FPF vector. The difference vectors were produced by subtracting the TPF (FPF) results of the second radiologist from the TPF (FPF) results of computer classifier for each subject. Since the neural network classifier outputted results using different threshold levels, the tests were applied for each threshold level. For each test, a null hypothesis which claimed the mean of the difference TPF (FPF) vector was statistically different than 0 within a confidence interval of 95% that was used. This null hypothesis aims to test the statistical equality of the results of the second radiologist and the computer classifier within a confidence interval.

## 3. Results

**Figure 2:**
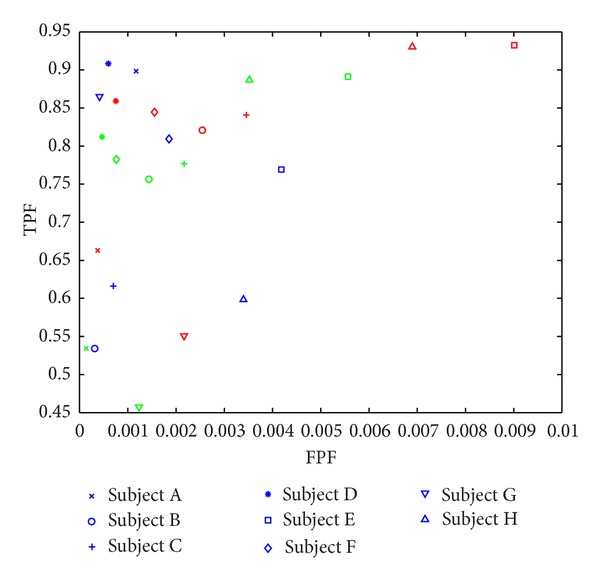
Scatter plot comparing the performances of the computer classifier versus secondary radiologist. Blue color denotes the performance comparison of second radiologist versus primary radiologist. Red color shows the performance comparison of computer classifier versus primary radiologist using threshold level of 59%. Green color shows the performance comparison of computer classifier versus primary radiologist using threshold level of 69%.

Figure 2 shows a scatter plot comparing the performance of the computer classifier with the second radiologist. The plot shows that the computer classifier can obtain at least as good true positive fraction (TPF) performance as the second radiologist at comparable false positive fraction (FPF) values.

Threshold value of 59% gave an overall TPF of 0.7786 and an overall FPF of 0.0042. Using threshold value of 69%, we obtained an overall TPF value of 0.6336 and an overall FPF value of 0.0020. The results obtained in this study using both threshold levels of 59% and 69% give a comparable result to the second radiologist who had an overall TPF value of 0.7350 and an overall FPF value of 0.0018. The null hypothesis for both TPF and FPF tests which compares the statistical significance was rejected while using the threshold values of 59% and 69% where the confidence interval was 95%.

**Figure 3:**
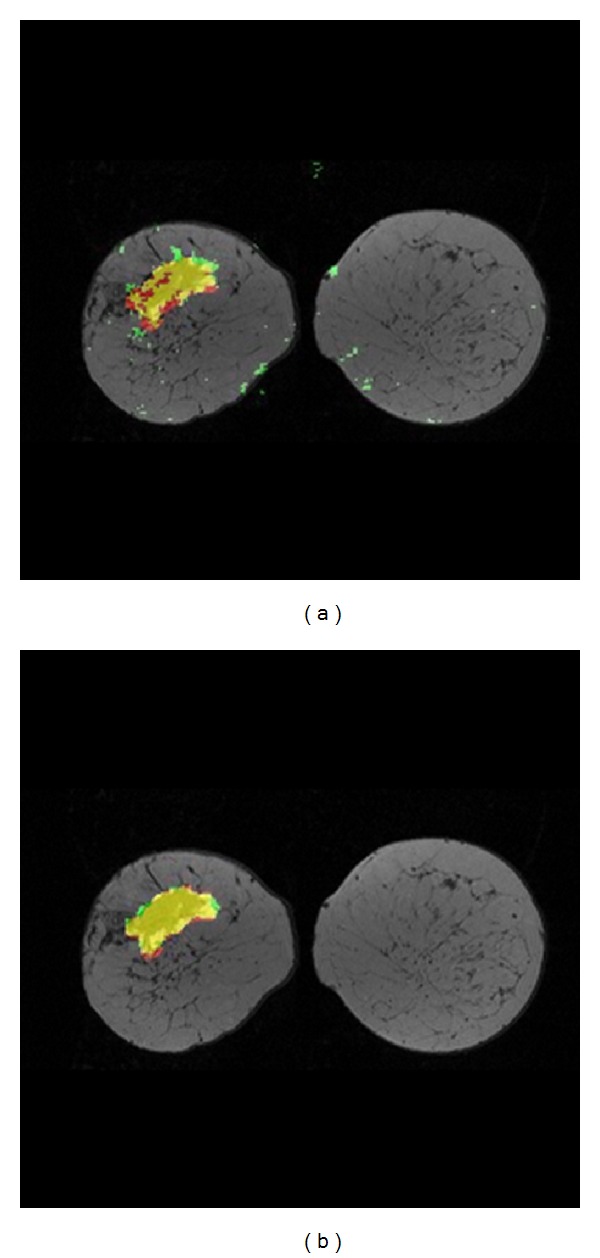
(a) Computer classifier versus primary radiologist at threshold level = 69%. (b) Second radiologist versus primary radiologist, subject D with IDC. Yellow = TP, green = FP, black/gray = TN, and red = FN.

Figure 3 shows a comparison of the computer classifier and the second radiologist for the experiments on subject D using a threshold value of 69%.  Figure 3(a) compares the computer classifier vs. primary radiologist, while Figure 3(b) compares the second radiologist vs. primary radiologist. In Figure 3(a), we can see some of the intramammary veins which are classified as malignant tissues as well.

**Figure 4:**
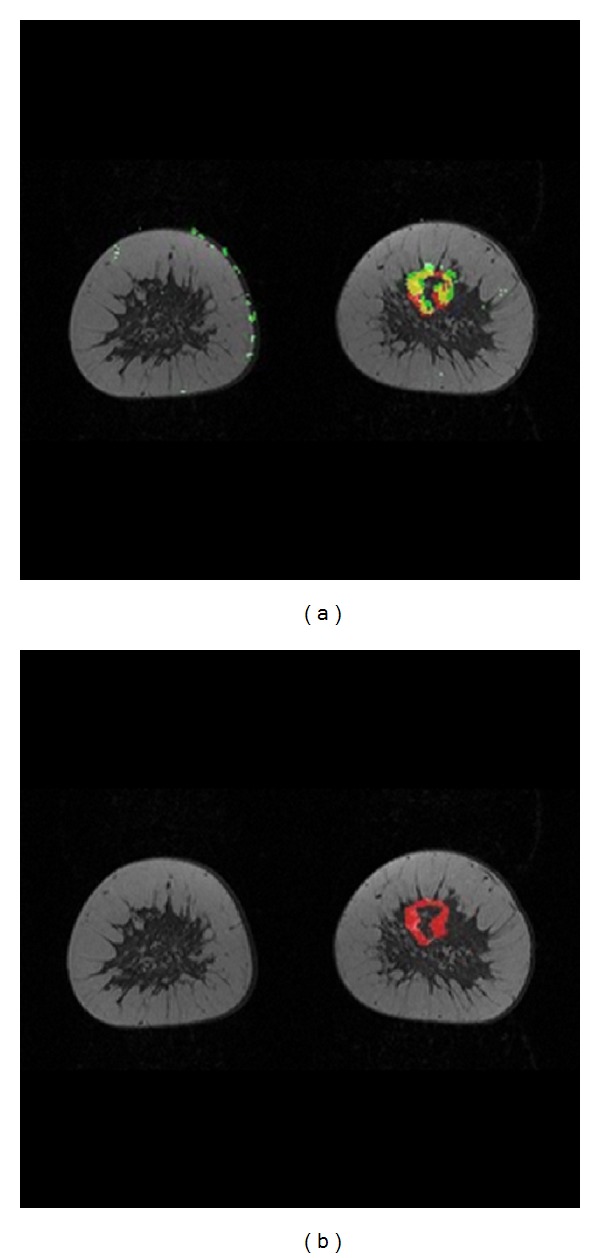
(a) Computer classifier versus primary radiologist at threshold level = 69%. (b) Second radiologist versus primary radiologist (a disagreement between radiologists is clearly shown), subject C with IDC. Yellow = TP, green = FP, black/gray = TN, and red = FN.

Figure 4 shows the results of the experiments implemented on subject C. In Figure 4(a), computer classifier performance compared to the base radiologist using a threshold level of 69% is displayed. The classifier also labeled some of the patient motion artifacts on the edge of the breast tissue to be malignant.  Figure 4(b) shows a major decision difference happening between the radiologists. A lesion in the slice is considered as a malignant tissue by the base radiologist; however, is considered to be benign by the second radiologist. The major reason behind the disagreement shown it the Figure 4(b) is the difficulty of through-plane assessment.

## 4. Discussion

With the rejection of the null hypothesis where the results are statistically significant, we can conclude that in comparison with the second radiologist, using threshold levels of 59% and 69%, the computer classifier had TPF and FPF mean values among the eight subjects that could not be statistically distinguished from the second radiologist within a confidence interval of 95% using a one-sample *t*-test.

The main advantage of the classifier is its ability to include all of the possible malignant lesions within the imaging volume. Most of the contradictions between the classifier and the base radiologist occur in the boundaries of the lesions. The computer did not miss a malignant tumor because in a SVOI, it clusters the appropriate combinations of *A*, *k*_ep_, and *k*_el_ that are characteristics of malignant tissue. Thus, this tool may be used to concentrate a reader's attention to potential malignant regions where the radiologist can use expertise to decide the appropriate classification (malignant or benign) of the mass and to redraw the region of interest (ROI) if necessary. 

The shown lesions seem to be quite simple to segment where a simple threshold on enhancement might be proposed; however, this proposal is not very appropriate because in DCE-MR image sequences, patient motion creates changes of voxel values which may be included in the thresholded segments. Thus, it is imperative to model the dynamics of a malignant tissue.

Sometimes, the computer classifier labels veins, patient motion, and other artifacts as malignant tissue, but can be adjusted by changing the threshold level of the neural network output. Increasing the threshold level of the neural network output decreases the artifacts; however, it also decreases the number of voxels correctly classified as malignant especially in the boundaries.

Using 3D SVOI, our CAD tool uses through-plane information in addition to in-plane information. Radiologists mentally connect through-plane information; hence, their assessments are based mostly on 2D coronal information. We have introduced a 3-D assessment where our CAD tool can help the radiologist to identify out-of-plane tumors connected between slices.

For the texture analysis, rather than analyzing postcontrast MR images as the previous studies reported in this paper, we have focused on DCE-MR parameter pseudoimages based on the two-compartment model which allowed us to study the dynamics of the tissues. The application of statistical image texture analysis to classify the local statistics of modeling parameters has not been previously reported in the medical imaging literature. We have used a feedforward neural network and as Twellmann suggested [20], we have used texture features for classification.

In conclusion, in this paper, we have demonstrated that using statistical texture analysis on parametric images can be used as a CAD tool that may be used to better facilitate quantitative tumor evaluation from DCE-MRI.

## References

[1] Knopp MV, Giesel FL, Marcos H, Von Tengg-Kobligk H, Choyke P (2001). Dynamic contrast-enhanced magnetic resonance imaging in oncology. *Topics in Magnetic Resonance Imaging*.

[2] Knopp MV, Weiss E, Sinn HP (1999). Pathophysiologic basis of contrast enhancement in breast tumors. *Journal of Magnetic Resonance Imaging*.

[3] Varini C, Degenhard A, Nattkemper TW Histologic characterization of DCE-MRI breast tumors with dimensional data reduction.

[4] Tourassi GD (1999). Journey toward computer-aided diagnosis: role of image texture analysis. *Radiology*.

[5] Lerski RA, Straughan K, Schad LR, Boyce D, Bluml S, Zuna I (1993). VIII. MR image texture analysis—an approach to tissue characterization. *Magnetic Resonance Imaging*.

[6] Kovalev V, Petrou M (1996). Multidimensional co-occurrence matrices for object recognition and matching. *Graphical Models and Image Processing*.

[7] Kovalev VA, Petrou M, Bondar YS (1999). Texture anisotropy in 3-D images. *IEEE Transactions on Image Processing*.

[8] Kovalev VA, Kruggel F, Gertz HJ, Von Cramon DY (2001). Three-dimensional texture analysis of MRI brain datasets. *IEEE Transactions on Medical Imaging*.

[9] Gibbs P, Turnbull LW (2003). Textural analysis of contrast-enhanced MR images of the breast. *Magnetic Resonance in Medicine*.

[10] Principe JC, Euliano NR, Lefebvre WC (2000). *Neural and Adaptive Systems: Fundamentals Through Simulations*.

[11] Vergnaghi D, Monti A, Setti E, Musumeci R (2001). A use of a neural network to evaluate contrast enhancement curves in breast magnetic resonance images. *Journal of Digital Imaging*.

[12] Lucht REA, Knopp MV, Brix G (2001). Classification of signal-time curves from dynamic MR mammography by neural networks. *Magnetic Resonance Imaging*.

[13] Lucht R, Delorme S, Brix G (2002). Neural network-based segmentation of dynamic MR mammographic images. *Magnetic Resonance Imaging*.

[14] Lucht REA, Delorme S, Hei J (2005). Classification of signal-time curves obtained by dynamic magnetic resonance mammography: Statistical comparison of quantitative methods. *Investigative Radiology*.

[15] Tzacheva AA, Najarian K, Brockway JP (2003). Breast cancer detection in gadolinium-enhanced mr images by static region descriptors and neural networks. *Journal of Magnetic Resonance Imaging*.

[16] Szabó BK, Aspelin P, Wiberg MK (2004). Neural network approach to the segmentation and classification of dynamic magnetic resonance images of the breast: comparison with empiric and quantitative kinetic parameters. *Academic Radiology*.

[17] Szabo BK, Wiberg MK, Bone B, Aspelin P (2004). Application of artificial neural networks to the analysis of dynamic MR imaging features of the breast. *European Radiology*.

[18] Twellmann T, Lichte O, Nattkemper TW (2005). An adaptive tissue characterization network for model-free visualization of dynamic contrast-enhanced magnetic resonance image data. *IEEE Transactions on Medical Imaging*.

[19] Haralick RM, Shanmugam K, Dinstein I (1973). Textural features for image classification. *IEEE Transactions on Systems, Man, and Cybernetics*.

[20] Brix G, Semmler W, Port R, Schad LR, Layer G, Lorenz WJ (1991). Pharmacokinetic parameters in CNS Gd-DTPA enhanced MR imaging. *Journal of Computer Assisted Tomography*.

[21] Kuhl CK, Mielcareck P, Klaschik S (1999). Dynamic breast MR imaging: Are signal intensity time course data useful for differential diagnosis of enhancing lesions?. *Radiology*.

[22] Collins D, Padhani A (2004). Dynamic magnetic resonance imaging of tumor perfusion: approaches and biomedical challenges. *IEEE Engineering in Medicine and Biology Magazine*.

[23] Sonka V, Hlavac M, Boyle R (1999). *Image Processing, Analysis, and Machine Vision*.

[24] Conners RW, Harlow CA (1980). A theoretical comparison of texture algorithms. *IEEE Transactions on Pattern Analysis and Machine Intelligence*.

[25] Hastings S, Kurc T, Langella S, Catalyurek U, Pan T, Saltz J Image processing for the grid:a toolkit for building grid-enabled image processing applications.

[26] Rosner B (2000). *Fundamentals of Biostatistics*.

